# Floristic Studies in the Light of Biodiversity Knowledge and Conservation

**DOI:** 10.3390/plants12162973

**Published:** 2023-08-17

**Authors:** Robert Philipp Wagensommer

**Affiliations:** Faculty of Education, Free University of Bozen-Bolzano, 39042 Brixen-Bressanone, Italy; robertphilipp.wagensommer@unibz.it

Floristic studies are often considered “simply” traditional research. However, the knowledge of the flora of a territory is essential to slow down the effects of anthropic pressure on biodiversity and essential for most of the research activities of modern botany, such as molecular research on the taxonomy of critical genera, which requires in-depth floristic knowledge for the correct identification of the analyzed taxa. Management actions, planning strategies, and biodiversity conservation are efficient and effective only with an in-depth knowledge of how many and which taxa constitute the floristic richness of a territory. Therefore, floristic research has not only been important in the past, but still has an important role in botanical studies today, even in countries with a long tradition of floristic studies and where the flora is considered already well known. Indeed, even in these countries, new taxa are still found [1,2] or described as new to science [3,4,5].

Although they play a key role in ecosystem functioning and human life, plants are generally overlooked by policy makers and humans in general [6]. An important task of flora experts is the correct identification of plants. This is even more important today, considering the increase in biologists unable to recognize even common plants, and the dwindling number of plant experts (i.e., botanists) able to identify plants [7].

In light of these considerations, eight research articles [8,9,10,11,12,13,14,15] and two reviews [16,17] were published in the journal *Plants*, in the Special Issue “Floristic Studies in the Light of Biodiversity Knowledge and Conservation”.

The eight research articles cover different taxonomic groups from Europe and Asia. Perrino et al. [8] evaluated the effects of ecology and climate on the composition of the essential oils of two species of wild officinal plants of the Lamiaceae family from Mediterranean Italy, i.e., *Satureja cuneifolia* Ten. and *Thymus spinulosus* Ten., the latter endemic to southern Italy and Sicily. The authors also presented a survey of the vegetation in which the two species grow. The results suggest a potential use of both species as food, pharmacy, cosmetics and perfumery. Glišić et al. [9] investigated the floristic composition and diversity of seven urban habitat types in 24 Serbian cities with different climatic characteristics. Significant differences were observed in habitats based on diagnostic species and representation of life forms, demonstrating that habitat type influences species composition much more than climate. Molnár et al. [10] presented a survey of the Orchidaceae diversity in Azerbaijani cemeteries, highlighting that Azerbaijani cemeteries can be important refuges for rare and threatened orchids. Furthermore, *Epipactis turcica* Kreutz was reported for the first time for the flora of Azerbaijan. Dar et al. [11] has published an analysis of the vegetation composition and distribution of *Aeluropus lagopoides* (L.) Trin. ex Thwaites communities in five different regions of Saudi Arabia, with a floristic survey, emphasizing environmental factors influencing species distribution in highly saline ecosystems such as Sabkhas. The authors concluded that, given the economic potential of *Aeluropus lagopoides* as a forage plant and sand stabilizer, the conservation of its habitats is of great importance, adding that this grass could be integrated as a promising forage candidate in saline-affected areas, even in the dry season. Apostolova et al. [12] presented a survey on the vascular and cryptogamic floristic diversity of ancient mounds in Bulgaria, demonstrating that as many as 98% of the more than one thousand vascular plant taxa recorded were native species. In addition, the lichen *Arthopyrenia salicis* A.Massal. was reported for the first time from Bulgaria. Iamonico [13] explored the plant diversity of a protected area in the city of Rome, reporting the inventory of the vascular flora, with information on its biological, ecological and biogeographical composition, and notes of the physiognomy of vegetation and landscape. Furthermore, *Denisophytum bessac* (Choiv.) E.Gagnon & G.P.Lewis has been reported as an alien species new to Europe. Bartolucci et al. [14] confirmed the presence of *Ranunculus gracilis* E.D.Clarke in Italy, highlighting the morphological characteristics that distinguish it from similar species, and assessing its conservation status in Italy. They concluded that floristic research and the study of herbarium collections are of crucial importance in the conservation of vascular plant biodiversity, and are necessary to collect data to plan the correct conservation strategies. Bonsanto et al. [15] has published a survey on the rare and little known species *Scabiosa garganica* Porta & Rigo ex Wettst., with a morphological analysis on the populations of the Gargano, including the *Locus classicus* of the species, and an ecological characterization. The new morphological framework has made it possible to highlight the taxonomic autonomy of the species and has helped to clarify its relationship with the related species *Scabiosa holosericea* Bertol. and *S. taygetea* Boiss. & Heldr. Furthermore, a lectotype was designated for the name *Scabiosa garganica*, and the conservation status of the species was assessed.

The two reviews deal with plants from different geographical regions, namely America and Africa on one side, and the Mediterranean Basin on the other. Gómez-Maqueo and Gamboa-deBuen [16] presented the biological diversity of the genus *Ceiba* (Malvaceae), that includes 18 recognized species, distributed in America and Africa, and introduced in various countries, especially in Asia, due to their ornamental interest and potential uses for their fiber. The authors pointed out that *Ceiba* species are also considered potentially useful for restoring ecosystems affected by human activity. On the other hand, Accogli et al. [17] presented the halophytic species traditionally gathered in south-eastern Italy, with particular regard to their ecology and distribution, traditional uses, medicinal properties, commercialization and first cultivation attempts.

In conclusion, it is possible to state with certainty that even today floristic studies are of great importance, especially in geographic areas in which the flora is not well known [18], in protected areas [19], and in biodiversity hotspots [20], but also in territories where the flora is well known [21], where these studies still contribute to the knowledge and conservation of biodiversity.

## References

[1] Wagensommer R.P., Fröhlich T., Fröhlich M. (2014). First record of the southeast European species *Cerinthe retorta* Sibth. & Sm. (Boraginaceae) in Italy and considerations on its distribution and conservation status. Acta Bot. Gall. Bot. Lett..

[2] Wagensommer R.P., Bartolucci F., Fiorentino M., Licht W., Peccenini S., Perrino E.V., Venanzoni R. (2017). First record for the flora of Italy and lectotypification of the name *Linum elegans* (Linaceae). Phytotaxa.

[3] Perrino E.V., Silletti G.N., Erben M., Wagensommer R.P. (2018). *Viola cassinensis* subsp. *lucana* (Violaceae), a new subspecies from the Lucanian Apennine, southern Italy. Phyton.

[4] Wagensommer R.P., Venanzoni R. (2021). *Geranium lucarinii* sp. nov. and re-evaluation of *G. kikianum* (Geraniaceae). Phytotaxa.

[5] Turco A., Medagli P., Wagensommer R.P., D’Emerico S., Gennaio R., Albano A. (2022). A morphometric study on *Ophrys* sect. *Pseudophrys* in Apulia (Italy) and discovery of *Ophrys japigiae* sp. nov. (Orchidaceae). Plant Biosyst..

[6] Balding M., Williams K.J.H. (2016). Plant blindness and the implications for plant conservation. Conserv. Biol..

[7] Crisci J.V., Katinas L., Apodaca M.J., Hoch P.C. (2020). The End of Botany. Trends Plant Sci..

[8] Perrino E.V., Valerio F., Jallali S., Trani A., Mezzapesa G.N. (2021). Ecological and Biological Properties of *Satureja cuneifolia* Ten. and *Thymus spinulosus* Ten.: Two Wild Officinal Species of Conservation Concern in Apulia (Italy). A Preliminary Survey. Plants.

[9] Glišić M., Jakovljević K., Lakušić D., Šinžar-Sekulić J., Vukojičić S., Tabašević M., Jovanović S. (2021). Influence of Habitat Types on Diversity and Species Composition of Urban Flora—A Case Study in Serbia. Plants.

[10] Molnár V. A., Löki V., Verbeeck M., Süveges K. (2021). Orchids of Azerbaijani Cemeteries. Plants.

[11] Dar B.A., Assaeed A.M., Al-Rowaily S.L., Al-Doss A.A., Abd-ElGawad A.M. (2022). Vegetation Composition of the Halophytic Grass *Aeluropus lagopoides* Communities within Coastal and Inland Sabkhas of Saudi Arabia. Plants.

[12] Apostolova I., Sopotlieva D., Valcheva M., Ganeva A., Shivarov V., Velev N., Vassilev K., Terziyska T., Nekhrizov G. (2022). First Survey of the Vascular and Cryptogam Flora on Bulgaria’s Ancient Mounds. Plants.

[13] Iamonico D. (2022). Biodiversity in Urban Areas: The Extraordinary Case of Appia Antica Regional Park (Rome, Italy). Plants.

[14] Bartolucci F., De Santis E., Conti F. (2022). Nomenclatural Synopsis, Revised Distribution and Conservation Status of *Ranunculus gracilis* (Ranunculaceae) in Italy. Plants.

[15] Bonsanto D., Biscotti N., Wagensommer R.P. (2023). New Morphological, Distribution, and Ecological Data on *Scabiosa garganica* (Caprifoliaceae), a Poorly Known Species of the Italian Flora, with Evaluation of Its Conservation Status and Typification of the Name. Plants.

[16] Gómez-Maqueo X., Gamboa-deBuen A. (2022). The Biology of the Genus *Ceiba*, a Potential Source for Sustainable Production of Natural Fiber. Plants.

[17] Accogli R., Tomaselli V., Direnzo P., Perrino E.V., Albanese G., Urbano M., Laghetti G. (2023). Edible Halophytes and Halo-Tolerant Species in Apulia Region (Southeastern Italy): Biogeography, Traditional Food Use and Potential Sustainable Crops. Plants.

[18] Figueiredo E., Smith G.F., César J. (2009). The Flora of Angola: First Record of Diversity and Endemism. Taxon.

[19] Jiménez J.E., Juárez P., Díaz A. (2016). Checklist of the vascular flora of Reserva Biológica San Luis, Costa Rica. Check List.

[20] Noroozi J., Naqinezhad A., Talebi A., Doostmohammadi M., Plutzar C., Rumpf S.B., Asgarpour Z., Schneeweiss G.M. (2019). Hotspots of vascular plant endemism in a global biodiversity hotspot in Southwest Asia suffer from significant conservation gaps. Biol. Conserv..

[21] Turco A., Albano A., Medagli P., D’Emerico S., Wagensommer R.P. (2023). Orchidaceae in Puglia (Italy): Consistency, Distribution, and Conservation. Plants.

